# Autoimmune Hemolytic Anemia as an Initial Manifestation of Splenic Marginal Zone Lymphoma

**DOI:** 10.7759/cureus.87506

**Published:** 2025-07-08

**Authors:** Neeti Nachammai K M, Vengadakrishnan K, Suja Lakshmanan, Saajid Anwar

**Affiliations:** 1 General Medicine, Sri Ramachandra Institute of Higher Education and Research, Chennai, IND; 2 Internal Medicine, Sri Ramachandra Institute of Higher Education and Research, Chennai, IND

**Keywords:** autoimmune hemolytic anemia (aiha), hemolytic anemia, non-hodgkin lymphoma, splenic marginal zone lymphoma, warm autoimmune hemolytic anemia

## Abstract

Autoimmune hemolytic anemia (AIHA) is caused by autoantibodies that target and destroy the individual's own red blood cells (RBCs). Warm AIHA is due to antibodies that are active at body temperature and are the most common type of AIHA. Herein, we report a 49-year-old woman who presented with breathlessness, easy fatiguability, and palpitations with blood picture showing severe anemia requiring multiple blood transfusions, leucocytosis, Direct Coombs Test (DCT) positivity, and elevated lactate dehydrogenase (LDH). The patient later developed increasing leukocytosis, for which bone marrow biopsy and immunophenotyping using flow cytometry were done, confirming the presence of underlying splenic marginal zone lymphoma resulting in secondary AIHA. The patient was referred to the hematology department and was started on treatment with steroids and rituximab.

## Introduction

Autoimmune hemolytic anemia (AIHA) is an immune-mediated destruction of red blood cells (RBCs), leading to anemia of varying severity. It may present as a primary disorder or a complication secondary to infections, autoimmune diseases, or lymphoproliferative disorders, such as chronic lymphocytic leukemia, autoimmune lymphoproliferative syndrome, and monoclonal gammopathy of undetermined significance (MGUS).

Splenic marginal zone lymphoma (SMZL) is a rare B-cell non-Hodgkin lymphoma that comprises less than 2% of all lymphomas, primarily involving the spleen and bone marrow [1,2]. While the classic presentation of SMZL includes mild cytopenia and splenomegaly, autoimmune manifestations such as AIHA can precede the diagnosis [3]. This case report highlights the importance of evaluating AIHA as a potential initial manifestation of SMZL.

## Case presentation

A 49-year-old woman presented to the outpatient department with complaints of breathlessness, easy fatiguability, exertional palpitations for one month, along with a 2 kg weight loss over one month. She had no history of fever, loss of appetite, bleeding manifestations, high-colored urine, night sweats, oral ulcers, multiple joint pains, or rash. There was no history of drug or toxin exposure, heavy menstrual bleeding, or irregular menstrual cycles.

She was initially evaluated at another hospital for severe anemia (Hb 4.2 g/dl) and was treated with three units of packed red blood cell (PRBC) transfusions and iron injections. Her peripheral smear showed normochromic normocytic anemia with leukocytosis and a positive Direct Coombs Test (DCT). Given persistent anemia despite multiple transfusions, the patient was referred to our center for further management.

On examination, the patient was conscious and oriented, and afebrile with a heart rate of 116/min, BP: 110/70 mmHg. Pallor and icterus were present. There was no lymphadenopathy, pedal edema, or clubbing. Abdominal examination revealed a palpable spleen 2 cm below the left costal margin. Other systemic examinations were unremarkable.

Laboratory investigations showed severe macrocytic anemia (Hb 3.8 g/dl), reticulocytosis, leucocytosis, indirect hyperbilirubinemia, elevated lactate dehydrogenase (LDH) 1558 (measured at 1:10 dilutions), and 3+ positive DCT (Table 1). Antinuclear antibody (ANA) and anti-double-stranded DNA (anti-dsDNA) were negative. A peripheral smear showed a low RBC count with macrocytic RBCs with anisopoikilocytosis, spherocytosis, agglutination, tear drop cells, and lymphocytic leukocytosis, features consistent with AIHA with lymphocytic leukocytosis. In view of severe anemia, she was transfused two units of PRBC on days 1 and 3. Abdominal ultrasonography revealed splenomegaly (14.6 × 5 cm) with normal echotexture.

**Table 1:** Laboratory investigations on day 1 Hb: hemoglobin; MCV: mean corpuscular volume; MCH: mean corpuscular hemoglobin; RBC: red blood cell; TLC: total leukocyte count; PLT: platelet count; TIBC: total iron-binding capacity; LDH: lactate dehydrogenase; DCT: Direct Coombs Test; SGOT: serum glutamic-oxaloacetic transaminase; SGPT: serum glutamic-pyruvic transaminase; BUN: blood urea nitrogen; Na: sodium; K: potassium; Cl: chloride; HBsAg: hepatitis B surface antigen; HCV Ab: hepatitis C virus antibody; ANA by IF: antinuclear antibody by immunofluorescence; anti-dsDNA titer: anti-double-stranded DNA titer

Laboratory investigations	Day 1	Reference range
Hb	3.8 g/dl	12-15 g/dl
MCV	111.6 FL	83-101 FL
MCH	30.4 pg	27-33 pg
RBC	1.12 mill/cumm	3.8-4.8 mill/cumm
TLC	25730 cells/cumm	4000-11000 cells/cumm
PLT	1.74 lakh/cumm	1.5-4.5 lakh/cumm
Serum iron	162 mcg/dl	60-180 mcg/dl
TIBC	289 mcg/dl	168-585 mcg/dl
LDH	1558 (1 in 10 dilution)	135-214
DCT	3+ positive	-
Indirect bilirubin	2.5 mg/dl	0.1-1 mg/dl
Direct bilirubin	0.84 mg/dl	<0.2 mg/dl
SGOT	79 IU/L	<32 IU/L
SGPT	19 IU/L	<33 IU/L
Albumin	3.8 g/dl	3.9-4.9 g/dl
Globulin	6.2 g/dl	2-3.5 g/dl
BUN	11 mg/dl	6-20 mg/dl
Serum creatinine	0.8 mg/dl	0.5-0.9 mg/dl
Na	130 mmol/L	136-145 mmol/L
K	4.1 mmol/L	3.5-5.1 mmol/L
Cl	97 mmol/L	98-107 mmol/L
Bicarb	22 mmol/L	22-29 mmol/L
Uric acid	10.2 mg/dl	2.4-5.7 mg/dl
Calcium	8.2 mg/dl	8.6-10 mg/dl
HIV p24, HIV 1&2 Ab	Non-reactive	-
HBsAg	Non-reactive	-
HCV Ab	Non-reactive	-
ANA by IF	Negative	-
dsDNA titer	<10 IU/ml	-

In view of suspected hemolytic anemia, intravenous methylprednisolone 125 mg once daily was initiated for five days, following which hemoglobin was found to be in an increasing trend (Table 2). Due to lymphocytic leukocytosis, a chronic lymphoproliferative disorder (CLPD) panel was performed using peripheral blood flow cytometry. Bone marrow biopsy revealed a lymphoproliferative disorder involving the marrow. Bone marrow aspiration showed hypercellular marrow with suppressed erythropoiesis and granulopoiesis and increased lymphocytes. Flow cytometry showed atypical lymphocytes identified in the SD45SS CD45 bright population. These cells were clonal, positive for lambda light chains, and negative for kappa. They expressed CD20, CD19, CD38, and CD200, while being negative for CD5 and CD10.

**Table 2:** Trend of complete blood count (CBC) during hospital stay

CBC	One month prior to admission	Day 1	Day 2	Day 3	Day 5	Day 8 discharge	Reference range
Hemoglobin	4.2 g/dl	3.8 g/dl	4.9 g/dl	4.8 g/dl	5.5 g/dl	7.7 g/dl	12-15 g/dl
Total leucocyte count	13000 cells/cumm	25730 cells/cumm	21130 cells/cumm	24900 cells/cumm	21440 cells/cumm	18250 cells/cumm	4000-11000 cells/cumm
Platelet	1.62 lakh/cumm	1.74 lakh/cumm	1.59 lakh/cumm	1.7 lakh/cumm	1.72 lakh/cumm	1.75 lakh/cumm	1.5-4.5 lakh/cumm
-	-	nRBCs17/100	Atypical lymphocytes 62	Atypical lymphocytes 48	Atypical lymphocytes 56	-	-

A final diagnosis of autoimmune hemolytic anemia secondary to SMZL was made. The patient was referred to the hematology department, where treatment was initiated with intravenous rituximab 600 mg.

## Discussion

AIHA is caused by the autoantibody-mediated destruction of self-red blood cells. Warm AIHA, the most common type, involves antibodies that are active at body temperature. Primary AIHA occurs without an underlying condition, whereas secondary AIHA is associated with infections (HIV, Epstein-Barr virus, hepatitis C virus {HCV}), autoimmune diseases (e.g., systemic lupus erythematosus, rheumatoid arthritis, ulcerative colitis), or lymphoproliferative disorders such as lymphoma, chronic lymphocytic leukemia (CLL), monoclonal gammopathy of undetermined significance (MGUS), and autoimmune lymphoproliferative syndrome [4]. Patients usually present with anemia, icterus, splenomegaly, reticulocytosis, DCT positive, elevated LDH, and peripheral smear showing spherocytes or microspherocytes. In adults with no obvious cause of hemolysis, flow cytometry is essential to detect low-grade lymphoproliferative disorders. First-line treatment includes glucocorticoids and rituximab, which is preferred in symptomatic patients and has shown better response rates than glucocorticoids alone [5,6].

The incidence of SMZL is approximately twice as high in White populations compared to others, though it occurs globally [7]. It is associated with autoimmune conditions that promote B-cell activation, such as Sjögren’s disease, systemic lupus erythematosus, hemolytic anemia, pernicious anemia, asthma, and chronic HCV infection [8]. Most patients present with splenomegaly, lymphocytosis, and cytopenias. While majority are asymptomatic, some may have symptoms related to splenomegaly or cytopenia. SMZL typically expresses IgM and IgD, along with B-cell antigens CD19 CD20 CD22, and Bcl2. CD5 negativity helps distinguish SMZL from CLL and mantle cell lymphoma [9,10]. Asymptomatic patients or those without complications are observed initially because they have a reasonably long survival [11]. However, patients with autoimmune complications require specific treatment. The presence of AIHA in patients with SMZL confers poor prognosis and serves as a marker of disease activity [12]. Rituximab is the preferred first-line agent for the treatment of SMZL compared with splenectomy or rituximab plus chemotherapy [13].

## Conclusions

This case highlights the importance of investigating for an underlying lymphoproliferative disorder in patients with autoimmune hemolysis. Given the poor prognosis associated with AIHA in SMZL, early identification and prompt initiation of targeted therapy are crucial.
